# Jacalin-Activated Macrophages Exhibit an Antitumor Phenotype

**DOI:** 10.1155/2016/2925657

**Published:** 2016-03-29

**Authors:** Cláudia Danella Polli, Luciana Pereira Ruas, Luciana Chain Veronez, Thais Herrero Geraldino, Fabiana Rossetto de Morais, Maria Cristina Roque-Barreira, Gabriela Pereira-da-Silva

**Affiliations:** ^1^Programa de Pós-Graduação em Imunologia Básica e Aplicada, FMRP/USP, 14049-900 Ribeirão Preto, SP, Brazil; ^2^Departamento de Biologia Celular e Molecular e Bioagentes Patogênicos, FMRP/USP, 14049-900 Ribeirão Preto, SP, Brazil; ^3^Faculdade de Ciências Farmacêuticas de Ribeirão Preto, FCFRP/USP, 14040-903 Ribeirão Preto, SP, Brazil; ^4^Departamento de Enfermagem Materno-Infantil e Saúde Pública, EERP/USP, 14040-902 Ribeirão Preto, SP, Brazil

## Abstract

Tumor-associated macrophages (TAMs) have an ambiguous and complex role in the carcinogenic process, since these cells can be polarized into different phenotypes (proinflammatory, antitumor cells or anti-inflammatory, protumor cells) by the tumor microenvironment. Given that the interactions between tumor cells and TAMs involve several players, a better understanding of the function and regulation of TAMs is crucial to interfere with their differentiation in attempts to skew TAM polarization into cells with a proinflammatory antitumor phenotype. In this study, we investigated the modulation of macrophage tumoricidal activities by the lectin jacalin. Jacalin bound to macrophage surface and induced the expression and/or release of mainly proinflammatory cytokines via NF-*κ*B signaling, as well as increased iNOS mRNA expression, suggesting that the lectin polarizes macrophages toward the antitumor phenotype. Therefore, tumoricidal activities of jacalin-stimulated macrophages were evaluated. High rates of tumor cell (human colon, HT-29, and breast, MCF-7, cells) apoptosis were observed upon incubation with supernatants from jacalin-stimulated macrophages. Taken together, these results indicate that jacalin, by exerting a proinflammatory activity, can direct macrophages to an antitumor phenotype. Deep knowledge of the regulation of TAM functions is essential for the development of innovative anticancer strategies.

## 1. Introduction 

Tumors are composed, besides the tumor cells, of a wide population of leukocytes and other types of infiltrating immune cells [1]. Immune cell infiltrates have been shown to significantly affect the malignant transformation [2] so that the interactions between immune and neoplastic cells are crucial in determining the course of tumorigenesis. Tumor-associated macrophages (TAMs) constitute a key component of the leukocytic infiltrate in tumors [3] and can exert different properties depending on the microenvironment. These cells act as fundamental inflammatory orchestrators in the development of different types of tumors.

Although TAMs are generally associated with tumor promoting roles in human cancers [4, 5], in certain types such as colorectal, stomach, and skin cancers, their presence correlates with good prognosis [6, 7]. TAMs are plastic cells that can exhibit a continuum of phenotypes [3, 8–10] and were characterized as M1 and M2 cells that represent the extremity of proinflammatory and anti-inflammatory phenotypes. Highly microbicidal and tumoricidal M1 macrophages are induced by IFN-*γ* alone or in concert with microbial stimuli, such as LPS or cytokines (e.g., TNF-*α*). These cells secrete high levels of proinflammatory cytokines (e.g., TNF-*α*, IL-12, IL-23, IL-6, and IL1-*β*), chemokines, and effector molecules, such as inducible nitric oxide synthase (iNOS) and MHCI/II. In contrast, immunosuppressive M2 macrophages are induced by IL-4 or IL-13 and are able to facilitate tumor progression. M2 macrophages express or secrete anti-inflammatory molecules, such as IL-10, TGF-*β*, and arginase-1 [10]. Given that TAMs can respond to different signals from the tumor microenvironment, there has been considerable effort to develop immunotherapies that skew their behavior to become cancer-suppressive.

Lectins, carbohydrate-binding proteins of nonimmune origin, can recognize aberrant glycosylation, a characteristic of all types of experimental and human cancers [11]. In cancer research, lectins were initially used as tumor recognition tools to differentiate malignant from benign tumors and the degree of glycosylation associated with metastasis [12]. In the last years, some plant lectins have been described for their potential therapeutic applications. Mistletoe lectins, ricin, and wheat germ agglutinin (WGA) have shown prominent antiproliferative and apoptosis-inducing activities toward cancer cells [13–17]. Besides, binding of lectins such as Concanavalin A and* Polygonatum cyrtonema* to glycosylated receptors on cancer cell surface has been shown to trigger autophagic cell death [18–21]. The number of studies evaluating the antitumor potential of plants lectins has increased, and some are at the preclinical and clinical levels [22–24].

Jacalin, a noncytotoxic plant lectin extracted from the seeds of* Artocarpus integrifolia*, was first described as a general T cell mitogen [25] that modulates Th1/Th2 cytokine secretion [26] by binding CD45 on T cell surface. Jacalin also induces interferon-*γ* and IL-6 secretion by monocytes [27]. This lectin specifically recognizes human IgA [28–30], D-galactose, and Thomsen-Friedenreich antigen (Gal*β*1-3GalNAc) [31], which is expressed in more than 85% of human carcinomas. In a recent study using mouse and rat bladder cancer models, jacalin showed strong discrimination between normal urothelium and neoplastic urothelium [32]. Due to the ability of jacalin to recognize TF antigens, its use as a carrier protein in cancer treatment has been considered [33–36]. Jacalin has been identified as a suitable molecule to carry nanoparticles conjugated with anticancer biomolecules and to selectively deliver these conjugates to tumor cells [37, 38]. TF-specific lectins are gaining clinical implications as they are increasingly known to inhibit cancer cell proliferation and metastasis in TF expressing cells [39].

In the present study, we analyzed the modulation of macrophage tumoricidal activities by jacalin. We show that the lectin drives macrophage polarization toward a proinflammatory, antitumor phenotype.

## 2. Materials and Methods

### 2.1. Monocyte Purification and Generation of Monocyte-Derived Macrophages

Human mononuclear cells were isolated from EDTA anticoagulated blood obtained from healthy volunteers who signed informed consent forms. An initial centrifugation for 20 minutes at 120 g allowed recovering the platelet-rich plasma, which was further centrifuged for 10 minutes at 1000 g to remove platelets. Blood cells were resuspended in the platelet-poor plasma and monocytes isolated by one step density-gradient centrifugation on Ficoll-Hypaque according to the manufacturer's instructions. Monocytes were seeded (10^6^ per mL), and after 2 h nonadherent cells were removed by aspiration. Next day adherent cells were treated with 100 ng/mL of PMA for 24 h. Cells were maintained in RPMI during 6 days until completely differentiated.

### 2.2. Purification and Biotinylation of Jacalin

**Figure 1:**
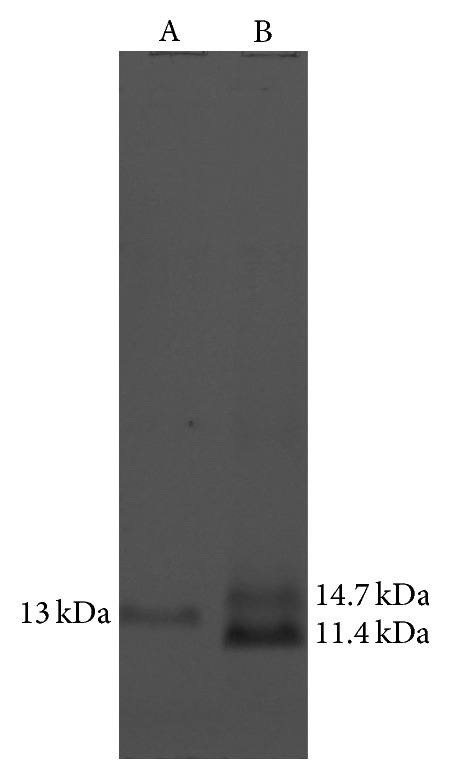
Electrophoretic analysis of purified jacalin. Jacalin, which was purified by affinity chromatography on immobilized D-galactose agarose, was analysed by polyacrylamide gel electrophoresis (SDS-PAGE) 12%. Lane A: Artin M (10 *μ*g); lane B: jacalin (10 *μ*g).

The lectin jacalin was purified by affinity chromatography on immobilized D-galactose agarose. The lectin purity was analyzed by sodium dodecyl sulfate polyacrylamide gel electrophoresis (SDS-PAGE) (Figure 1), and protein concentration was determined by the bicinchoninic acid (BCA) assay (Pierce). Jacalin was biotinylated using Sulfo-NHS-LC-Biotin (Pierce) according to the manufacturer's recommendations.

### 2.3. Binding Assay

Macrophages, HT-29 cells, and MCF-7 cells (10^6^/mL) were incubated with biotinylated jacalin (20 *μ*g/mL) for 30 min at 4°C. For some of the assays the lectin was previously incubated with 100 mM D-galactose for 30 min at 37°C. After incubation with streptavidin-Alexa-488 for 30 min at 4°C, cells were fixed with 1% formaldehyde and analyzed by flow cytometry. Results were analyzed using the software FACSDiva.

### 2.4. MTT Assay

Cell proliferation was analyzed using the 3-(4,5-dimethylthiazol-2-yl)-2,5-diphenyltetrazolium bromide (MTT) assay. Briefly, cells were seeded in 96-well plates (1 × 10^4^ cells/well) and were incubated with different concentrations of jacalin (2.5–40 *μ*g/mL) or LPS (1 *μ*g/mL) for 24–72 h. Supernatants from jacalin-stimulated macrophages were also tested. After washing twice with PBS, 20 *μ*L of MTT solution (0.125 mg/mL) was added to the wells. After incubation for 4 h at 37°C, the resultant formazan crystals were dissolved in acid-isopropanol (50% of isopropanol, 0.4% of chloridric acid at 37%) containing triton 5% (100 *μ*L) and the absorbance intensity was measured by a microplate reader at 490 nm with a reference wavelength of 570 nm. All experiments were performed in triplicate, and the relative cell viability (%) was expressed as a percentage relative to the untreated control cells.

### 2.5. Enzyme-Linked Immunosorbent Assays (ELISAs)

Macrophages were incubated with jacalin (2.5 to 40 *μ*g/mL) or LPS (1 *μ*g/mL) for 48 h. The supernatants were harvested and the concentrations of TNF, IL-6, IL-12p70, IL-10, IL-1*β*, and TGF-*β* were measured by ELISA, according to the manufacturer's instructions (BD Biosciences).

### 2.6. Real-Time Quantitative PCR

For determination of relative mRNA expression, we utilized the 2^−ΔΔCT^ method. Total RNA was extracted with TRIzol (Invitrogen, Carlsbad, CA, United States) according to the manufacturer's instructions. Reverse transcription was performed with oligo dT primers. Real-time polymerase chain reaction (PCR) was carried out in a StepOnePlus Real-Time PCR System (Applied Biosystems, Foster City, CA, USA) with SYBR Green PCR Master Mix (Promega). Gene expression levels were quantified relative to the expression of *β*-actin. Primers used for Real-Timer PCR (5′-3′) were as follows: *β*-actin, F-TGACGGGGTCACCCACACTGTGCCCATCTA, R-CTAGAAGCATTGCGGTGGACGATGGAGGG; TNF-*α*, F-GGAAGAGAACCTGCCTGGC, R-GGAAGAGAACCTGCCTGGC; IL-1*β*, F-CAAGGCACAACAGGCTGCT,  R-CATTTCACTGGCGAGCTCAG; MIP-1*α*, F-CCGACCGCCTGCTGCTTCA,  R-CTGCCGGCTTCGCTTGGTTAG; CXCL8, F-AGGGTTGCCAGATGCAATAC,  R-AAACCAAGGCACAGTGGAAC; CCL2, F-CAGCCAGATGCAATCAATGCC,  R-TGGAATCCTGAACCCACTTCT; iNOS, F-TCCGAGGCAAACAGCACATTCA,  R-GGGTTGGGGGTGTGGTGATGT; TRAIL, F-CTTCACAGTGCTCCTGCAGT,  R-TTAGCCAACTAAAAAGGCCCC.

### 2.7. NF-*κ*B Activation

We used RAW 264.7 cell line, which expresses the luciferase reporter gene under the transcriptional control of an NF-*κ*B response element. Briefly, 5 × 10^5^ cells/wells were stimulated with jacalin (20 or 40 *μ*g/mL) or LPS (1 *μ*g/mL) for, respectively, 4 hours or 1 hour (LPS) at 37°C. After washing with PBS, cells were incubated with 40 *μ*L of cell lysis buffer containing Tris-HCl pH 8.5, EDTA 5 mM, NaCl 200 mM, and triton 1% during 20 min at 4°C. Cell lysates were collected and centrifuged at 20000 ×g for 8 min at 4°C and 10 *μ*L of supernatants was collected. After addition of substrate solution (luciferase assay system kit, Promega), luciferase activity was determined using a GloMax-Multi Jr (Promega) luminometer.

### 2.8. Apoptosis Assay

HT-29 and MCF-7 cells were cultured with supernatants from jacalin-activated macrophages for 72 hours. Cells were rinsed twice with PBS, harvested, and stained with Annexin V-FITC/Propidium Iodide (PI), according to the manufacturer's instructions (BD Biosciences). Apoptosis was determined by fluorescence-activated cell sorting (FACSCanto, Becton Dickinson, CA, USA). The percentage of apoptotic cells (Annexin V-PI-positive) was calculated using the software FACSDiva.

### 2.9. Statistics

Results are expressed as the mean ± standard error of the mean (SEM). The differences between treatments were analyzed by Student's *t*-test. Statistical analyses were performed using GraphPad Prism 5 software (La Jolla, CA, USA). The differences were indicated as ^*∗*^ *p* ≤ 0,05, ^*∗∗*^ *p* ≤ 0,01, and ^*∗∗∗*^ *p* ≤ 0,001 compared to control cells.

## 3. Results

### 3.1. Purified Jacalin Has 11.4 and 14.7 kDa Protein Bands

Jacalin samples were submitted to reducing SDS-PAGE. After electrophoresis, purified jacalin showed two protein bands of apparent molecular weights of 11.4 and 14.7 kDa (Figure 1, lane B). Artin M, the other lectin from* A. integrifolia* which was used as the control of the purification procedure, showed a single protein band of apparent molecular weight of 13 kDa (Figure 1, lane A).

### 3.2. Jacalin Ligands Are Expressed on the Surface of Both Human Macrophages and Tumor Cells

**Figure 2:**
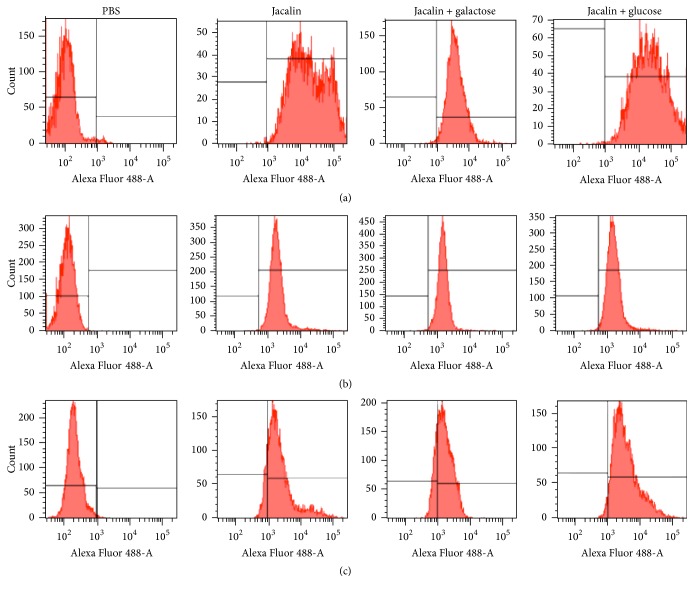
Jacalin binds to the surface of macrophages, HT-29 cells, and MCF-7 cells. Macrophages (a), HT-29 (b), or MCF-7 (c) cells (1 × 10^6^ cells) were incubated for 30 minutes at 4°C with biotinylated jacalin (20 *μ*g/mL) previously incubated with 100 mM D-galactose or glucose for 30 minutes at room temperature. After washing, cells were incubated with Alexa 488-conjugated streptavidin and analyzed by flow cytometry. Results are representative of three independent assays.

We studied jacalin binding to the surface of human macrophages and HT-29 and MCF-7 tumor cell lines. Cells were incubated with biotinylated jacalin followed by Alexa-488-streptavidin and then analyzed by flow cytometry. As shown in Figure 2, all cells (100%) were surface labeled. Based on these data, we investigated whether jacalin binding to the cell surface is dependent on carbohydrate recognition. Addition of galactose (specific sugar) inhibited jacalin binding to macrophages (85%), HT-29 cells (40%), and MCF-7 cells (62%). As expected, glucose (nonspecific sugar) did not affect binding. These results suggest that jacalin binds to macrophage and tumor cell surface mainly via its carbohydrate recognition domains (CRDs).

### 3.3. Effects of Jacalin on HT-29 and MCF-7 Cell Viability

**Figure 3:**
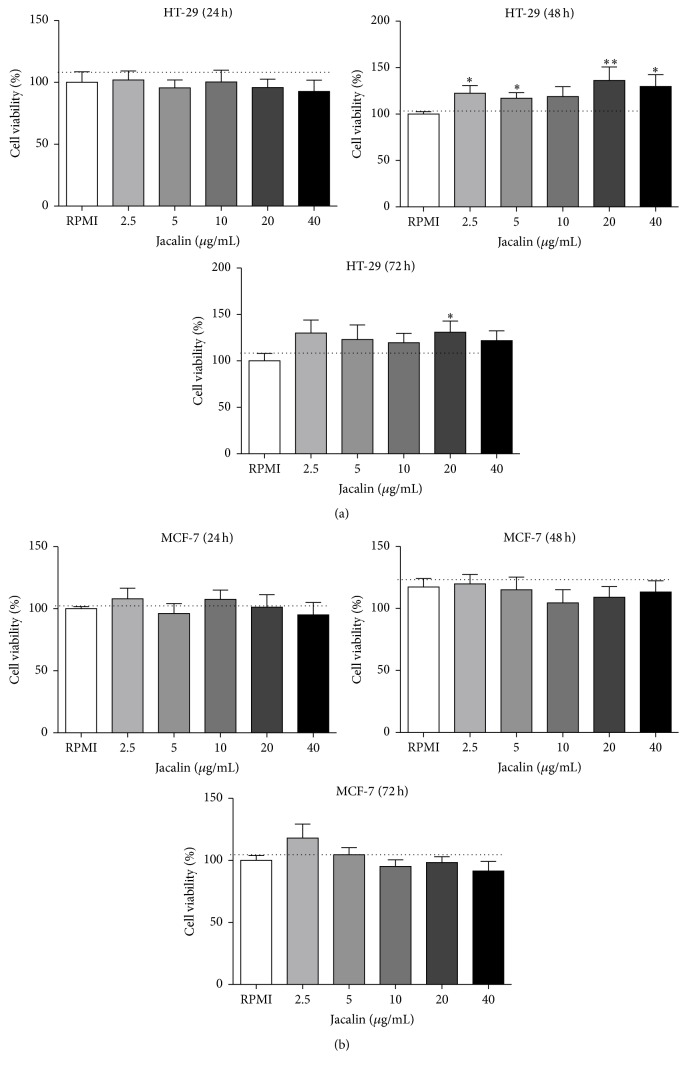
Effects of jacalin on HT-29 and MCF-7 cell viability. HT-29 (a) or MCF-7 (b) cells were incubated with jacalin (2.5 to 40 *μ*g/mL) during 24 to 72 h. After that, MTT solution was added to the cells and four hours later the solvent solution was added. Results represent the mean ± SEM of three independent experiments. ^*∗*^ *p* ≤ 0,05 and ^*∗∗*^ *p* ≤ 0,01 compared to control.

Since jacalin binds to HT-29 and MCF-7 cells, we performed MTT assays to investigate its potential cytotoxicity against these tumor cells. Cells were incubated with jacalin at the indicated concentrations for 24, 48, and 72 hours, and then cell viability was measured (Figure 3). Cell proliferation was not inhibited by any concentration of jacalin (Figure 3(a)). These results show that jacalin has no direct cytotoxic effect on these tumor cells.

### 3.4. Production of Mediators by Jacalin-Activated Macrophages

**Figure 4:**
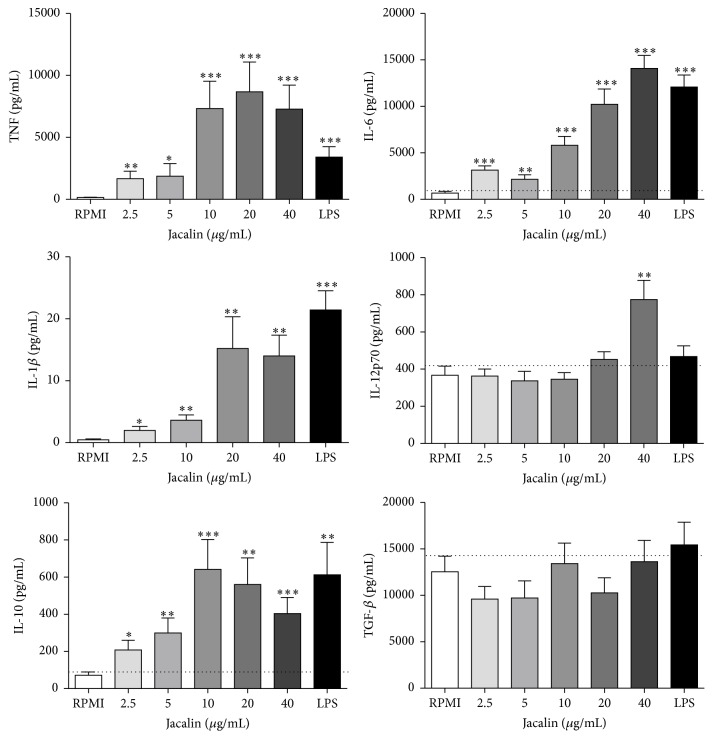
Production of anti-inflammatory or proinflammatory mediators by jacalin-stimulated macrophages. Human macrophages were stimulated with jacalin (2.5 to 40 *μ*g/mL) or LPS (1 *μ*g/mL) during 48 h at 37°C. The supernatant was collected and the levels of cytokines were determined by ELISA. Values represent the mean ± SEM of two independent experiments. ^*∗*^ *p* ≤ 0,05, ^*∗∗*^ *p* ≤ 0,01, and ^*∗∗∗*^ *p* ≤ 0,001 compared to control.

Next we investigated whether jacalin induces cytokine secretion by human macrophages. Macrophages were stimulated for 48 hours with jacalin (2.5 to 40 *μ*g/mL) or LPS (1 *μ*g/mL), and cytokine secretion was evaluated by ELISA (Figure 4). The lectin induced the production of high levels of TNF and IL-6. IL-6 secretion by jacalin-stimulated macrophages increased in a dose-dependent manner. The highest levels of TNF and IL-1*β* were obtained with 10 to 40 *μ*g/mL of jacalin. Only the highest concentration (40 *μ*g/mL) of jacalin induced IL-12p70 release. We also detected IL-10 in the supernatant of jacalin-stimulated macrophages. The lectin did not affect the basal production of TGF-*β*.

### 3.5. Effects of Jacalin on the Expression of Tumoricidal Macrophage Mediators

**Figure 5:**
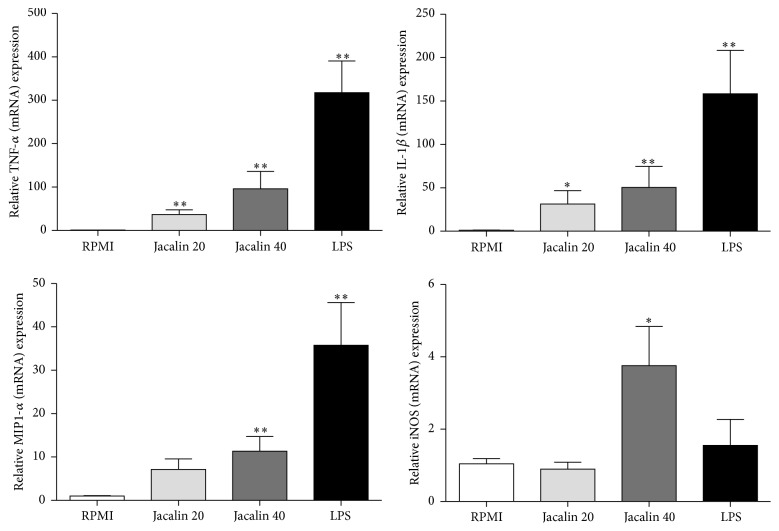
Effects of jacalin on the expression of mediators by macrophages. Macrophages were stimulated with jacalin (20 or 40 *μ*g/mL) or LPS (1 *μ*g/mL) during 4 h. Expression of TNF-*α*, IL-1*β*, MIP-1*α*, and iNOS mRNA was determined by real-time quantitative RT-PCR. Each column represents the mean ± SEM of *n* = 3 independent observations in three separate experiments. ^*∗*^ *p* ≤ 0,05 and ^*∗∗*^ *p* ≤ 0,01 compared to control.

Next we studied the mRNA expression of mediators normally produced by M1 tumoricidal macrophages. RT-PCR analysis revealed a significant increase of TNF-*α*, IL-1*β*, MIP-1*α*, and iNOS mRNA levels in jacalin-stimulated macrophages (Figure 5). On the other hand, we did not detect changes in CXCL8, CCL2, and TRAIL (TNF-related apoptosis-inducing ligand) mRNA expression after cell stimulation (data not shown). These results support the hypothesis that jacalin drives macrophages toward the tumoricidal phenotype.

### 3.6. Jacalin Activates the NF-*κ*B Signaling Pathway in Macrophages

**Figure 6:**
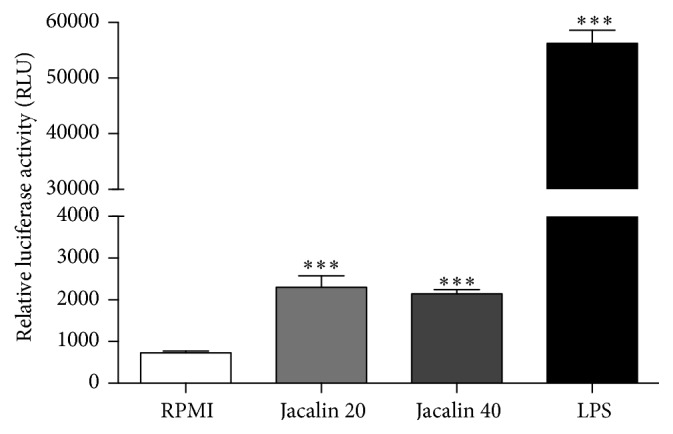
Jacalin-induced NF-*κ*B activation in RAW 264.7 cells. RAW luc cells were stimulated with jacalin (20 and 40 *μ*g/mL) during 4 h or LPS (1 *μ*g/mL) during 1 h. The cells were then harvested and luciferase activity was determined using a luciferase assay system kit. Data represent the mean ± SEM of two different assays. ^*∗∗∗*^ *p* ≤ 0,001 compared to control.

Given that NF-*κ*B activation is critically involved in the production of proinflammatory mediators, we investigated whether jacalin is able to activate this signaling pathway. RAW NF-*κ*B-luc cells were stimulated with jacalin (20 or 40 *μ*g/mL) or with LPS (1 *μ*g/mL) and NF-*κ*B activation was measured by luciferase activity. Both concentrations of jacalin induced similar levels of NF-*κ*B activation, as demonstrated by luciferase intensity (Figure 6). These results show that jacalin-induced macrophage activation involves the NF-*κ*B pathway.

### 3.7. Supernatants from Jacalin-Activated Macrophages Induce Tumor Cell Death

**Figure 7:**
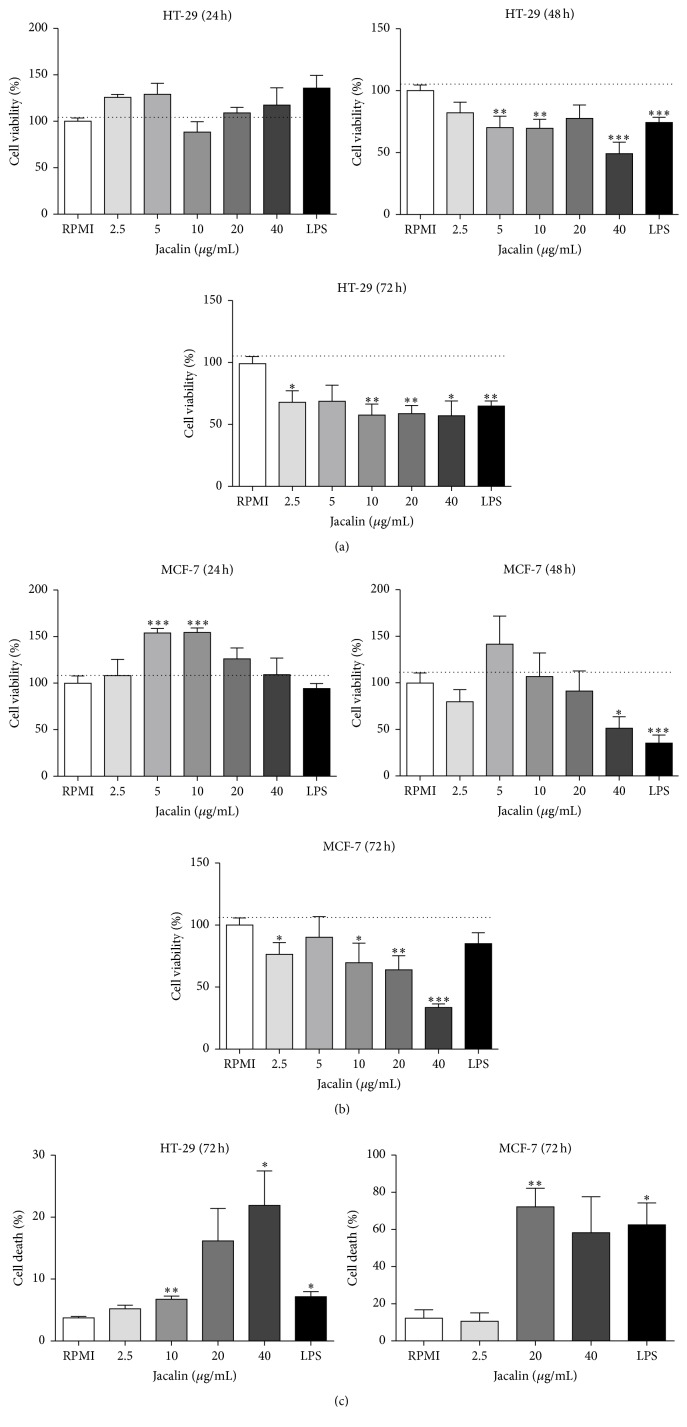
Effects of supernatants from jacalin-stimulated macrophages on HT-29 and MCF-7 cell viability. HT-29 or MCF-7 cells were incubated during 72 h with supernatants from macrophages stimulated with jacalin (2.5 to 40 *μ*g/mL) or LPS (1 *μ*g/mL) for 24 to 72 h. After that, MTT solution was added to the cells and four hours later the solvent solution was added (a and b), or cells were double stained with Annexin V-FITC and PI and analyzed by flow cytometry (c). Values represent the mean ± SEM of three independent experiments. ^*∗*^ *p* ≤ 0,05, ^*∗∗*^ *p* ≤ 0,01, and ^*∗∗∗*^ *p* ≤ 0,001 compared to control.

As jacalin-activated macrophages produced high amounts of proinflammatory mediators like TNF-*α*, we investigated whether the supernatant from these cells has a cytotoxic effect on tumor cell lines. HT-29 and MCF-7 cells were incubated with supernatants from jacalin-stimulated macrophages for 24, 48, or 72 hours, and cell proliferation was analyzed by MTT assays (Figures 7(a) and 7(b)). Tumor cell proliferation was not affected upon incubation with macrophage supernatants during 24 hours. After 48 hours, incubation of HT-29 cells with supernatants from macrophages stimulated with 5 and 10 *μ*g/mL of jacalin resulted in a 30% reduction of cell proliferation, while 40 *μ*g/mL of jacalin induced a 50% reduction. Similarly, the incubation of MCF-7 cells with supernatants from macrophages stimulated with 40 *μ*g/mL of jacalin decreased cell proliferation by 50%. Higher antiproliferative effects were observed after 72 h incubation with supernatants from jacalin-stimulated macrophages. In these conditions, we found a 32–43% decrease in HT-29 cell proliferation and 23–64% inhibition of MCF-7 cell proliferation.

Given the cytotoxic effects of supernatants from jacalin-stimulated macrophages on tumor cells, we performed flow cytometric analysis to assess whether the supernatants induce tumor cell death (Figure 7(c)). As demonstrated by the percentage of Annexin V-PI-positive cells, treatment of HT-29 cells with supernatants from macrophages stimulated with 10 and 40 *μ*g/mL of jacalin significantly increased tumor cell apoptosis. Similarly, incubation of MCF-7 cells with supernatants from macrophages stimulated with 20 *μ*g/mL of jacalin resulted in 80% of cell death. These results indicate that jacalin induces macrophages to produce soluble mediators that exert cytotoxic effects on tumor cells.

## 4. Discussion

In the present study, we analyzed the modulation of macrophage tumoricidal activities against human colon (HT-29) and breast (MCF-7) adenocarcinoma cells by the lectin jacalin. Our results show that jacalin polarizes macrophages toward a proinflammatory, antitumor phenotype.

Given that jacalin is able to recognize glycans like the TF antigen [31, 40], which is expressed on a large number of tumors, we investigated the binding of the lectin to HT-29 and MCF-7 cell surfaces. Our results suggest that jacalin binds to tumor cells via its carbohydrate recognition domains (CRDs) as well as through protein-protein interactions, as the binding was partially inhibited in the presence of the specific sugar. Although some studies have reported that jacalin has antiproliferative effects on HT-29 cells [41–43], we did not observe a direct cytotoxic effect of the lectin on either HT-29 or MCF-7 cells.

Within the tumor context, macrophages are increasingly recognized as pivotal regulators. By exerting ambiguous functions, these cells can significantly affect the course of tumor development [6, 44–47]. Depending on the cancer type, the higher density of TAMs is associated with poor or favorable prognosis [48–52]. In a recent study, the dense infiltration of CD40^+^ macrophages indicated a favorable prognosis in colorectal cancer patients [53].

In response to different stimuli, macrophages undergo polarized activation. While proinflammatory M1 macrophages exhibit tumoricidal activities, anti-inflammatory M2 macrophages are oriented to tumor progress [54–56]. In the present study, jacalin-stimulated macrophages produced the proinflammatory cytokines TNF, IL-6, IL-12, and IL-1*β*, as well as the anti-inflammatory cytokine IL-10. Furthermore, these cells showed increased expression of iNOS, a signature molecule for M1, and MIP-1*α* mRNA, a chemokine that amplifies the proinflammatory response by inducing the production of TNF-*α*, IL-6, and IL-1*β* by macrophages [57].

The NF-*κ*B transcription factor, a key regulator of carcinogenesis, can influence tumor progress both positively and negatively [58]. Despite its controversial role in tumor cells [58], its role in TAMs is well established. In the early stages of carcinogenesis, NF-*κ*B is activated in M1 TAMs (TNF-*α* ^high^, IL-1^high^, IL-12^high^, IL-10^low^, and TGF-*β* ^low^) in sites of preneoplastic lesions, exacerbating local inflammation. Tumor growth is paralleled by the gradual inhibition of NF-*κ*B in infiltrating macrophages, favoring a phenotypic switch toward M2 suppressive cells (TNF-*α* ^low^, IL-1^low^, IL-12^low^, IL-10^high^, and TGF-*β* ^high^) [4, 59, 60]. In this study, using RAW 246.7 cells constitutively expressing NF-*κ*B-dependent luciferase reporter gene (RAW luc), we observed that macrophage activation induced by jacalin involves the NF-*κ*B signaling pathway. The ability of jacalin to induce NF-*κ*B activation and the production of proinflammatory cytokines by macrophages supports the hypothesis that the lectin is a molecule that drives/skews TAM polarization toward a tumoricidal phenotype.

Although macrophages predominantly produced proinflammatory cytokines in response to jacalin stimulation, these cells also produced considerable amounts of IL-10. It is conceivable that these cells do not assume the classical M1 phenotype but, instead, polarize to an intermediate phenotype close to M1. These results may reflect the more complex situation that usually occurs in tumor microenvironment where diverse stimuli coexist and can be combined, inducing macrophages to polarize into phenotypes that differ in terms of cytokine production and expression of MHC and costimulatory molecules or receptors [61]. Indeed, in human and experimental tumors, macrophages have been shown to exhibit mixed phenotypes, expressing both M1 and M2 markers [6, 62]. Macrophages expressing high levels of both inducible nitric oxide synthase (M1 marker) and arginase-1 (M2 marker) have been described in mouse models of colon carcinoma and sarcoma [63]. Therefore, the distinction between M1 and M2 macrophages represents two extremes and does not reflect the total spectrum of phenotypes that macrophages can adopt [64].

Given that jacalin had no direct cytotoxic effects on tumor cells but induced macrophages to potentially express tumoricidal activities, we evaluated the effects of supernatants from jacalin-stimulated macrophages on tumor cell viability. Incubation of HT-29 and MCF-7 cells with the supernatants resulted in significant inhibition of cell proliferation and in cell death. We hypothesize that the high levels of proinflammatory mediators present in the supernatants, particularly TNF, induced tumor cell death. Indeed, TNF-*α* is known to induce tumor cell apoptosis [65]. However, jacalin was not able to induce TRAIL expression on macrophages, a known death ligand important for tumor killing [66]. Furthermore, jacalin stimulation increased MIP-1*α* mRNA expression, a molecule that can further amplify the proinflammatory response induced by jacalin. In agreement with our results, Dumont and coworkers (2008) have shown that the supernatants from LPS-activated, therefore M1-like macrophages, contained high levels of proinflammatory cytokines such as GM-CSF, IL-1*β*, IL-6, IL-8, and TNF-*α* and exhibited growth inhibitory activities on human colon adenocarcinoma cells [54].

In a recent study by our group using a mouse model of chemically induced colon carcinogenesis, we showed that tumor-bearing animals treated with jacalin produced increased levels of proinflammatory cytokines such as IL-1*β*, TNF, IL-12, and IFN-*γ*. The proinflammatory activity exerted by jacalin was associated with decreased proliferation and increased apoptosis of tumor cells (Geraldino et al., submitted). These results corroborate those found in the current investigation, suggesting that jacalin exerts proinflammatory, antitumor activities.

Because targeting macrophages either by ablation or repolarization toward the M1 phenotype may potentiate cancer therapy, TAMs have been increasingly considered as potential targets for antitumor therapy [67–72]. However, therapeutic approaches targeting these cells may have systemic toxicities, as they will also affect macrophages outside the tumor microenvironment. Therefore, it is imperative to find molecules that are capable of targeting specifically TAM. Based on the fact that jackfruit seeds are edible and form part of the diet in the tropics [73, 74], the fact that jacalin is specific for the cancer-associated Thomsen-Friedenreich (T) carbohydrate antigen and, as we show here, the fact that this lectin is able to activate macrophages toward a tumoricidal phenotype, jacalin would be a suitable candidate for adjuvant cancer therapy. Considering the complex interplay between TAMs and tumor cells, a better understanding of the regulation of protumor or antitumor functions of macrophages is essential for the development of innovative anticancer strategies.
